# Epigenomics in Understanding Racial Disparities of Alzheimer’s Disease and Related Dementias

**DOI:** 10.3390/ijms27020739

**Published:** 2026-01-11

**Authors:** Kumudu Subasinghe, Harlan P. Jones, Robert Barber, Nicole Phillips

**Affiliations:** 1 Department of Microbiology, Immunology and Genetics, UNT Health, 3500 Camp Bowie Blvd., Fort Worth, TX 76107, USA; harlan.jones@unthsc.edu (H.P.J.); nicole.phillips@unthsc.edu (N.P.); 2 Institute for Health Disparities, UNT Health, 3500 Camp Bowie Blvd., Fort Worth, TX 76107, USA; 3 Department of Family Medicine & Manipulative Medicine, Texas College of Osteopathic Medicine, UNT Health, 3500 Camp Bowie Blvd., Fort Worth, TX 76107, USA; robert.barber@unthsc.edu; 4 Institute for Translational Medicine, UNT Health, 3500 Camp Bowie Blvd., Fort Worth, TX 76107, USA

**Keywords:** Alzheimer’s disease (AD), AD and related dementias (ADRDs), racioethnic disparities in AD, epigenomics, racial disparities in AD, precision medicine

## Abstract

Alzheimer’s disease (AD) and related dementias (ADRD) are neurodegenerative conditions that cause gradual deterioration of cognition, memory and language in the elderly. AD has been declared as a health priority by the World Health Organization (WHO) considering its severity and unavailability of a permanent cure. Although the global AD/ADRD population is made up of many ethno-racial groups, the majority of AD studies have focused on the Caucasian population. The few AD studies conducted on minority populations in the US have found that significant AD health disparities exist, demonstrating that African Americans and Hispanics have a significantly higher prevalence of AD and related dementias, with their risk often approaching twice that of White individuals. For the past few years, epigenomic research has played an important role in understanding health disparities among diverse racial and ethnic groups. Unlike genetic studies, which focus on the DNA sequence that one is born with, epigenomics investigates how changes in gene expression due to extrinsic environmental exposures may impact disease pathophysiology. Recent epigenomic studies appear to be promising in not only understanding disease pathology but also in developing diagnostic and therapeutic tools for AD with population specificity. However, there is only a handful of studies and review articles available addressing the epigenomic applications in irradicating racial disparities in AD/ADRD. Therefore, the aim of this review is to discuss the recent findings of epigenomic studies in AD and related dementias, their contribution in irradicating racioethnic disparities and insights into the future direction of their application in precision medicine.

## 1. Introduction

Alzheimer’s disease (AD) is a complex neurodegenerative disease that leads to progressive impairment of cognition, memory and language among the older population. Amyloid-β associated amyloid plaque and Tau phosphorylation in the brain are considered early indicators of AD onset, which is considered the primary cause of dementia [1]. People with underlying diseases, including diabetes, cardiovascular disease and hypertension, have higher risk of AD at later stages of their lives, as the risk of AD increases gradually with age for all individuals regardless of the presence of other risk comorbidities [2]. Dietary habits and lifestyle (e.g., behavior, psychical, etc.) also play major roles in risk of AD [2,3,4].

AD has been declared a “global health priority” by the World Health Organization due to the lack of permanent remedy and it being the sixth leading cause of death in the United States [5]. It is predicted that global new AD cases will increase by 10 million each year. The number of individuals aged 65 and older in the United States is expected to increase from 55 million in 2020 to 94 million by 2060. In 2022, approximately 6.5 million older Americans, nearly three-quarters of whom were over 75, were living with Alzheimer’s dementia. This number is expected to increase to 13.8 million by 2060 [6]. As the population ages, the prevalence of AD and related dementias (ADRDs) is also anticipated to rise significantly [6,7] which could tremendously burden the economy and health care sector due to the increased need of providing care for AD patients. The US AD population includes diverse racioethnic groups with significant health disparities in AD epidemiology [8]. Medical risk factors associated with AD such as hypertension, diabetes, obesity, depression have been found to be important indicators of racioethnic disparities in AD. Genetic, cerebrospinal fluid [CSF] and blood proteomics that have widely been used as AD biomarkers have significant variation among different racioethnic groups [9]. However, direct comparisons among large samples representing the diverse group of populations are limited both in the United States and global AD studies [10]. The “missing heritability” of AD which is referred to as the phenomenon where the heritability of a trait or disease cannot be entirely explained by the genetic variants identified through traditional genome-wide association studies (GWAS) [11], has been also been a limitation in population specific AD studies [12].

Epigenetic studies may play a key role in understanding health disparities among diverse groups understudied in the past few years. Epigenetics is the study of chemical alterations to DNA that affect the way genes are expressed, or “turned on,” and are necessary for proper mammalian maintenance and development. In contrast to genetic studies, which focus on the DNA sequence with which one is born with, epigenetic studies look at how changes in gene expression may be influenced in disease pathology. DNA methylation for an example is one of the epigenetic modifications commonly seen in AD due to various environmental factors. Some studies show differential global DNA methylation patterns in the entorhinal cortex and hippocampus of postmortem AD brains [13]. Therefore, understanding of epigenomics in AD may facilitate proper diagnostics, therapeutics, and interventions of the patients.

AD represents a specific neurodegenerative pathology characterized by amyloid-β plaques and tau neurofibrillary tangles, whereas dementia is a clinical syndrome encompassing multiple etiologies including AD, vascular dementia, Lewy body diseases and mixed pathologies. Importantly, most large population-based studies examining racial disparities rely on dementia diagnoses derived from clinical records or cognitive assessments rather than biomarker-confirmed AD. As a result, much of the epidemiological literature reflects ADRD rather than pathologically defined AD. Throughout this review, we distinguish between AD and ADRD and explicitly note when findings derive from dementia-based cohorts.

## 2. Methods

This narrative review was conducted by surveying peer-reviewed literature indexed in PubMed and Google Scholar between 2012 and 2025. The search terms included combinations of “Alzheimer’s disease”, “dementia”, “epigenetics”, “epigenomics”, “DNA methylation”, “histone modification”, “miRNA”, “racial disparities”, “ethnicity” and “social determinants of health”. Priority was given to human studies, population-based cohorts, postmortem brain studies, and well-characterized animal or cellular models where mechanistic insights were relevant. Given the limited number of studies explicitly examining epigenetics and race/ethnicity in AD and ADRD, relevant studies in dementia more broadly were included and critically appraised.

## 3. Racial Disparities in AD

Studies conducted in the US alone show several racial differences in AD implicating inequity in socioeconomic status, educational opportunities, psychosocial stressors like persistent discrimination, and higher rates of comorbidities such as cardiovascular disease and associated risk factors, collectively contributing to this health disparity [14]. For example, it is known that Hispanic individuals and African Americans have a 1.5 and 2 times elevated risk of AD, respectively, compared to their white counterparts. AD health disparities are not only population-specific but are also gender-specific. Studies show that women have a greater prevalence of ADRD than males across all races and ethnicities. Hispanic and African American females within the United States have higher incidence rates than males [15]. These findings show significant health disparities associated with AD pathology across different racioethnic groups in the United States and globally indicate that social and behavioral variables are key causes of these disparities.

Mayeda et al. (2016), conducted a survey using six racial and ethnic groups in the United States to identify racial disparities in dementia. This study analyzed a large sample set using electronic health records of older adult individuals in Northern California who have equal access to healthcare for over 14 years. This comparison includes African Americans, American Indians and Alaska Natives (AIAN), Asian Americans, Latinos, Pacific Islanders and Whites. The inequalities were analyzed based on both sex and age. The results indicates that African Americans and AIANs had the highest age-adjusted dementia incidence rates, Asian Americans had the lowest, while Latinos, Pacific Islanders, and Whites had intermediate rates. African Americans were at a higher risk than Asian Americans. At the age of 65, 38% African Americans, 35% AIANs, 32% Latino, 25% Pacific Islanders, 30% White, and 28% Asian-Americans had the cumulative 25-year risk for dementia [10].

Matthews et al.’s 2019 study is based on an assessment of the future US ADRD burden by age, gender, race and ethnicity. The research utilized subgroup-specific prevalence rates among Medicare fee-for-service beneficiaries aged 65 years and older in 2014 combined with population projection data from the US Census Bureau spanning 2015 to 2060. The findings include that nearly 5 million individuals aged over 65 in the United States were diagnosed with ADRD in 2014. ADRD was more prevalent in women than in men. The diagnosis rate was approximately 3.6 percent among individuals aged 65 to 74 years, 13.6 percent among those aged 75 to 84 years, and 34.6 percent among those aged 85 years and older. Among ethnic groups, Asians and Pacific Islanders had the lowest incidence of ADRD, followed by American Indians and Alaska Natives, non-Hispanic whites, individuals of mixed ethnicity, Hispanics, and blacks. At age 85, over 43 percent of blacks and 40 percent of Hispanics were projected to have the highest burden of ADRD-related disease [16].

By 2060, the anticipated growth percentages in total population by race and ethnicity are projected to be 75 percent for non-Hispanic whites, 172 percent for African Americans, 270 percent for Asian and Pacific Islanders, 274 percent for American Indians and Alaska Natives, and 391 percent for Hispanics [16,17]. It is foreseen that minority populations, referring to racial and ethnic groups other than non-Hispanic whites as classified by the US Census Bureau, will surpass the growth rate of the nonminority population in the coming decades. Consequently, the United States is expected to transition into a majority-minority nation by 2050. Notably, the prevalence of ADRD is highest among racial and ethnic groups projected to experience the most significant population growth. This underscores the need for increased research on ADRD, with a specific focus on minority groups, particularly those with higher rates of population growth [17].

## 4. Conceptual Framework and Historical Context of Epigenetics

Epigenetics refers to heritable yet potentially reversible modifications of gene regulation that occur without changes to the underlying DNA sequence. Originally conceptualized by Waddington to explain developmental plasticity, modern epigenetics encompasses DNA methylation, histone modifications, chromatin remodeling and non-coding RNAs [18]. While enthusiasm for epigenetics has expanded rapidly across biomedical research, it is critical to distinguish casual mechanisms from associative epigenomic signatures. As noted by Ospelt, the widespread adoption of epigenetic explanations necessitates careful interpretation, particularly when linking environmental exposures, ethnicity and disease risk. In this context, epigenetics is best viewed as a molecular interface through social, behavioral and environmental factors influence disease susceptibility rather than a deterministic explanation of racial differences [18,19].

## 5. Epigenomics of AD/ADRD

**Figure 1:**
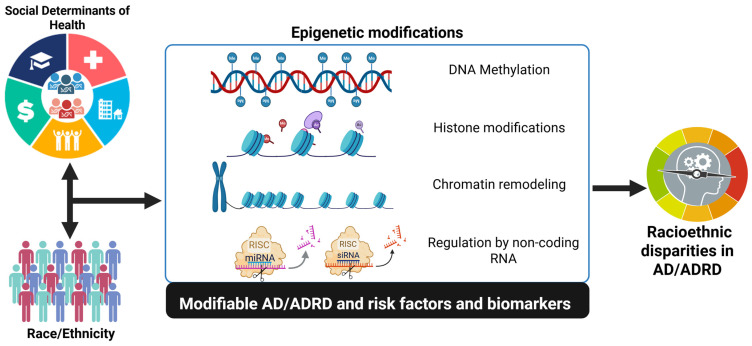
Schematic illustrating how social determinants of health interact with epigenetic mechanisms to influence AD and ADRD. Environmental and psychosocial exposures shape DNA methylation, histone modifications (acetylation, methylation, phosphorylation, ubiquitination), chromatin remodeling, and microRNA regulation. These epigenetic alterations affect neuronal, immune, and metabolic pathways, contributing to population-specific differences in ADRD risk and progression.

Social determinants of health, including chronic psychosocial stress, socioeconomic disadvantage, environmental toxin exposure, dietary patters and structural inequities can exert lasting biological effects through epigenetic mechanisms. Prolonged exposure to stressors such as discrimination and neighborhood deprivation has been associated with altered DNA methylation patters in genes involved in inflammation, metabolic regulation and stress responsiveness. These environmentally induced epigenetic changes may accumulate over the life course and interact with aging-related processes to influence susceptibility to AD and ADRD. Importantly, such mechanisms provide a biologically plausible framework linking social context to racioethnic disparities in disease risk and progression, without implying genetic determinism (Figure 1) [20,21]. In recent years, medical research has incr4easingly focused on social epigenomics to understand how environmental and social conditions influence human biology through epigenomic regulation and contribute to contemporary health inequalities [15]. Social epigenomics examines the biological effects of environmental and psychosocial stressors across the lifespan. Mancilla et al., 2020, discuss several social determinants of health, defined as the conditions of the environment where an individual lives, learns, works, plays, worships and ages, emphasizing their potential to shape long-term health outcomes through epigenetic pathways [15].

Several research studies suggest that both physical stressors and psychosocial experiences can leave epigenetic marks in the next generation. For example, offspring of some holocaust survivors have altered biochemistry and behavior. The Dutch famine that occurred during 1944–1945 resulted in women giving birth to children smaller in size. Those children were also susceptible to many diseases like diabetics, cardiovascular diseases and obesity [22]. According to Post, 2021, multiple studies have found shorter telomeres in Black individuals who have experienced racial discrimination, and this collection of environmentally induced epigenetic alterations in telomere length is a potential contributor to their greater sensitivity to numerous medical and psychiatric disorders [22].

With the increase of research funding and attention gained from the society, Alzheimer’s research has shown significant progress in modern diagnostic techniques. However, existing methods have been ineffective in distinguishing AD from other causes of dementia-related neuropathology. Biomarkers like decreased Aβ levels in cerebrospinal fluid (CSF) and the identification of Aβ deposits or tau accumulation in the brain through positron emission tomography (PET) imaging aid in assessing and monitoring the advancement of AD pathology [23,24,25]. A conclusive diagnosis of AD can only be made posthumously, requiring the identification of distinct amyloid plaques and neurofibrillary tangles in the brains of afflicted individuals. Therefore, precise early-stage diagnostics for Alzheimer’s are essential. Current research emphasizes epigenetic alterations as significant contributors to AD development, offering potential for the discovery of dependable diagnostic methods [26].

### 5.1. DNA Methylation

DNA Methylation is the most studied epigenetic modification [27] in AD. The DNA methyltransferase (DNMT) family of enzymes facilitates DNA methylation by adding a methyl group to the 5th atom of the cytosine ring. S-adenosyl methionine serves as the donor of the methyl group. DNA methylation at specific sites within a locus can act as either a repressive or activating signal for gene expression. In interaction with histone modifiers, DNA methylation can either prevent the transcription machinery from binding or create an environment that promotes transcription. Throughout development, the DNA methylation pattern in the genome changes due to a controlled balance between de novo methylation and demethylation. As a result, differentiated cells acquire a distinct DNA methylation pattern that fine-tunes tissue-specific gene expression [28]. Most DNA methylation takes place on cytosines that are followed by guanine bases, creating regions of DNA enriched in cytosine-phosphate-guanine motifs, known as CpG islands [26]. This type of DNA methylation is known as 5-methylcytosine (5-mC) [15]. CpG islands typically reside in regulatory regions of DNA preventing the transcriptional activation of the gene, thereby reduce gene expression [29]. While the addition of a methyl group to cytosine does not alter its base pairing, it can impact the binding of transcriptional activators to DNA. Methyl-CpG binding domain proteins (MBDs), belonging to a protein family, exhibit a preference for binding to methylated CpGs. Acting as anchors, MBDs facilitate the recruitment of additional proteins. DNA methylation is tissue-specific and serves as a crucial mediator of environmental cues, aiding cells in adapting to diverse environmental conditions while preserving their identity [26,30].

DNA methylation has been implicated in AD, and genome-wide DNA methylation analysis provides important information about varying DNA methylation patterns in disease pathologies [30]. Watson et al. (2016) performed a genome-wide analysis of DNA methylation in the superior temporal gyrus (STG) of 34 patients with AD and 34 control subjects. The STG is a brain region that has been shown to exhibit notable dysregulation of AD-associated genes. The researchers utilized the Illumina Infinium HumanMethylation450 array platform for their study [31]. Studies show that DNA methylation variability in genes can increase the disease susceptibility [30]. Huo et al. (2019) carried out a study to identify variably methylated probes (VMPs) and variably methylated regions (VMRs) in postmortem brain tissue to determine whether changes in DNA methylation stability play a role in AD pathology. VMPs represent single DNA methylation sites and VMR indicates multiple VMPs clustered together. Hu et al. have used specifically selected VMRs and VMPs that have association with AD pathology. They identified 249 VMPs that were clustered into 133 VMR regions that are associated with amyloid-β and 115 VMPs clustered into 14 VMRs that are associate with neurofibrillary tangles. Overall, their findings indicate that DNA methylation instability influences AD neuropathology and emphasized the importance of examining methylation variability in epigenetic studies [30].

DNA hydroxy methylation (5hmC) is another epigenetic marker [32] that is generated from the oxidized version of 5mC. Although the exact role of 5hmC is unknown [33], some studies show its dynamic interaction with transcription factors promoting positive regulation of gene expression, hence is also considered an intermediate product of active demethylation [34,35,36]. 5hmCs are also localized in enhancer regions of some genes promoting positive enhancer activity [33]. Recent discoveries suggest that 5hmC plays a role in the development of disorders such as AD as it appears that 5hmC is enriched in the brain [34] thus opening new avenues for treating the condition by correcting methylation and hydroxymethylation abnormalities. Moreover, the postmortem AD brains show differentially hydroxymethylated genes associated with AD pathology indicating the importance of studying 5hmC patterns in AD [32].

Coppieters et al. (2014), found a global hypermethylation in the AD brain, as well as significantly higher levels of 5hmC in AD human middle frontal gyrus (MFG) and middle temporal gyrus (MTG), with no noticeable impact of gender, age, postmortem delay, or tissue storage duration. They discovered using double-fluorescent immunolabeling that levels of 5mC and 5hmC were low in astrocytes and microglia but high in neurons in normal and AD brains. Moreover, the study shows that 5mC and 5hmC are also positively correlated with levels of amyloid beta (Aβ) plaques, neurofibrillary tangles (NFTs), and ubiquitin, indicating a connection to the severity of the pathology [37].

Mitochondrial dysfunction is found to be another epigenetic factor associated with neurological diseases. Mitochondrial dysfunction can lead to the production of reactive oxygen species (ROS), which can damage DNA and proteins, influencing epigenetic marks. Moreover, mitochondrial signals can interact with the nucleus to modulate transcription factors and other regulatory proteins, further affecting epigenetic regulation. Thus, mitochondrial dysfunction is intertwined with epigenetic mechanisms, influencing cellular function and potentially contributing to various diseases. Mitochondrial DNA is highly susceptible to oxidative stress. Various aspects of mitochondrial function, including mitochondrial morphology and quantity, oxidative phosphorylation, production of reactive oxygen species (ROS), calcium regulation, mitochondria-endoplasmic reticulum interactions, mitochondrial DNA integrity, biogenesis, mitophagy, and mitochondrial transport within neurons, have been identified to influence the AD brain [38,39,40,41].

Silzer et al. (2020) showed that methylation occurring at the 9th position (“p9 site”) of mitochondrial tRNAs (mt-tRNA) impacts translational efficiency and subsequent mitochondrial function. However, directly assessing mt-RNA methylation presents challenges. Recent advances in RNA sequence-based techniques have emerged in detecting post-transcriptional methylation. While research on p9 methylation has been conducted in healthy human populations and in cancer, its investigation in neurodegenerative diseases, where mitochondrial dysfunction is a prominent early feature, remains limited. Given the well-established association between mitochondrial dysfunction and neurodegenerative disease progression, their study provides insights into potential molecular mechanisms underlying this dysfunction [41]. They demonstrate a shared cerebellar pathogenesis in prevalent tauopathies like AD and progressive supranuclear palsy. Nonetheless, whether p9 hypermethylation serves as a cause or a consequence of pathology remains uncertain [42].

### 5.2. Histone Modification

Histone modifications encompass a range of alterations such as acetylation, phosphorylation, methylation, ubiquitination, ADP ribosylation, and sumoylation. Among these, histone acetylation and deacetylation play crucial roles in controlling gene transcription by modulating chromatin structure and accessibility to transcription factors. The main protein components of chromatin, alkaline histones, are abundant in the nuclei of eukaryotic cells. These histones form octamers that wrap around DNA to create nucleosomes, each containing two distinct copies of the four core histones: H2A, H2B, H3, and H4. Acetylation of histones H3 and H4 is recognized as a characteristic of chromatin in an “open” state. This acetylation, occurring primarily on lysine residues at the N-terminus of core histones, promotes gene transcription by neutralizing the positive charge of histone tails and reducing histone binding to negatively charged DNA. Consequently, histone acetylation is often associated with the activation of gene transcription, whereas histone deacetylation typically leads to the repression of gene transcription [43].

Histone ubiquitination is an important but less extensively studied epigenetic modification in AD. Ubiquitination of histones H2A and H2B influences chromatin compaction, transcriptional regulation, and DNA damage responses. Dysregulation of ubiquitin–proteasome pathways has been implicated in protein aggregation and impaired clearance mechanisms in AD, suggesting that altered histone ubiquitination may contribute indirectly to neurodegenerative processes [44,45,46]. Histone phosphorylation plays a critical role in DNA damage response, transcriptional activation, and neuronal signaling. Increased phosphorylation of histone H2AX (γH2AX), a marker of DNA double-strand breaks, has been observed in AD brains, reflecting heightened genomic stress. These changes may interact with other epigenetic modifications to influence neuronal vulnerability and disease progression [46,47].

Wan et al. (2016) utilized RNA-seq data and ChIP-seq histone modification data from CK-p25 AD model and control mice to identify 72 lncRNA genes alongside 4917 peaks of H3K4me3 and 1624 peaks of H3K27me3 displaying differential patterns between AD and control samples. Moreover, they observed that 92 peaks of histone modification H3K4me3 were located in the promoters of 39 differentially expressed lncRNA genes, while 8 peaks of histone modification H3K27me3 were positioned upstream of 7 differentially expressed lncRNA genes. These findings suggest that histone modification may play a significant role in the transcriptional regulation of the majority of lncRNA genes in AD [48].

### 5.3. Chromatin Remodeling

Chromatins in eukaryotic cells are a DNA packaging mechanism that are made out of proteins, DNA and RNA. Linear nuclear DNA is wrapped around histone proteins to make chromatin structure. Nucleosomes are the primary repeating unit of the chromatin structure. Nucleosome is an octameric disc composed of two copies of each of the four canonical histone proteins (H3, H4, H2A, and H2B). Chromatin remodeling is a dynamic, cell and environment-specific process that allows for regulation of DNA packaging and, as a result, access to transcriptional machinery at specified loci. It relies heavily on histone post-translational modifications and integrates a wide range of environmental stimuli to effect powerful and long-lasting alterations in gene expression [49]. The vast range of chromatin remodeling complexes available allow for a diversity of chromatin remodeling processes. Remodelers have the ability to slide nucleosomes, expel histone octamers, remove and replace H2A-H2B dimers, and replace dimers. These activities require the breakdown of all 14 histone-DNA interactions and require 12–14 kcal mol^−1^ [50,51].

Lee at al. (2020), conducted a study showing how H3K9me3-dependent heterochromatin remodeling might be affected and identified which H3K9me3-related epigenomic markers are linked to the development of sporadic AD. They conducted a combined analysis of H3K9me3-chromatin immunoprecipitation sequencing (ChIP-seq) and mRNA sequencing (mRNA-seq) on brain samples from both AD patients and healthy controls. Their network analysis revealed that epigenomic profiles influenced by H3K9me3 are connected to synaptic dysfunction. They further used qPCR on brain tissue samples from both normal and AD subjects to validate H3K9me3-enriched epigenomes indicating that changes in chromatin remodeling and gene expression associated with H3K9me3 play a role in the progression of AD [52].

### 5.4. Regulation by Non-Coding RNA

Most of the genetic material in humans does not encode proteins; instead, it is known as noncoding RNA (ncRNA). Originally dismissed as useless RNA, ncRNAs are now recognized for their role in controlling cellular processes and influencing gene expression and suppression. Various types of non-coding RNAs (ncRNAs), like microRNAs (miRNAs) and long non-coding RNAs (lncRNAs), have been identified, distinguished by factors such as the specific regions of the human genome they originate from and the length of their sequences [53].

miRNAs are a type of small non-coding RNA that regulate messenger RNA (mRNA) translation by binding to it complementarily, causing mRNA degradation or block protein expression. They are short strands with 20–25 base pairs. miRNA can be used as blood biomarkers as they can also be found in extracellular fluids and blood plasma. Due to their role in fine-tuning gene expression, miRNAs exhibit a highly dynamic and intricate regulation within the brain. Their expression levels can shift in as little as 90 min, and their lifespan in brain cells usually lasts no more than 3.5 h [54,55]. Due to their compact size, amphipathic properties, and excellent solubility, miRNAs exhibit high mobility and are widespread throughout the brain and central nervous system [56]. They stand out as the smallest nucleic acid signaling molecules known in eukaryotes. Currently, approximately 2650 miRNAs have been identified and rigorously studied in humans [57].

Numerous studies have unveiled the potential of miRNAs in AD pathology. Beyond DNA methylation, miRNA-mediated regulation of mitochondrial gene networks has been increasingly implicated in AD, linking epigenetic control of energy metabolism, oxidative stress responses and neuronal vulnerability to disease progression [58,59]. Recently, there’s been a notable increase in interest regarding vesicle-encapsulated or biofluid-enriched monomeric miRNAs. These molecules are being explored as potential RNA markers that could serve as predictive or diagnostic biomarkers for detecting and tracking the advancement of AD [57,58]. Neuronal-enriched extracellular vesicles have gained particular attention as a source of brain-derived miRNA detectable in peripheral biofluids, with emerging evidence demonstrating disease-associated alterations that support their utility as minimally invasive biomarkers for AD and ADRD [60,61].

A study by Lee et al. (2019) found that circular RNAs (circRNAs) have an impact on the function of miRNAs, suggesting that the circRNA–miRNA–mRNA network could play a role in disease development. Consequently, researchers have examined the dysregulated circRNAs in the Tg2576 mouse model of AD, their potential influence on downstream target mRNAs, and their significance in the progression of the disease [62]. They found that the dysregulation of circRNAs was associated with an increase in the dysregulation of relevant downstream target mRNAs [54]. While transgenic mouse models such as Tg2576 provide valuable insight into amyloid-associated molecular mechanisms, they do not recapitulate the full spectrum of human AD pathology, including tau pathology, neurodegeneration and cognitive heterogeneity. Accordingly, findings from these models should be interpreted as hypotheses-generating rather than direct representations of human AD or its racioethnic disparities [63].

Nagaraj et al. (2019)’s review states that among the 137 miRNAs found to be altered in AD blood, 36 have been consistently replicated in independent studies, while 13 out of 166 miRNAs identified as differential in AD cerebrospinal fluid (CSF) have been frequently observed. Only three miRNAs have consistently shown alterations across three studied specimens: blood, CSF, and brain (hsa-miR-146a, hsa-miR-125b, hsa-miR-135a) [64]. Nevertheless, all 36 frequently divergent miRNAs in AD blood hold promise as components of a diagnostic panel. Such a miRNA panel, given their projected functions, could unveil various pathways contributing to AD pathogenesis, potentially leading towards precision medicine. Additionally, the same study revealed a significant overlap between dysregulated miRNAs in AD and those implicated in cancer. However, the direction of miRNA alterations in cancer and AD often contrasts, suggesting an epigenetic trade-off between these two disorders [64,65].

To summarize representative examples of miRNAs implicated in AD and ADRD, including their biological localization and reported functional associations, a summary table is provided below (Table 1).

## 6. Applications of Epigenomics in Understanding Racial Disparities in AD

Population-associated epigenetic alterations implicated in AD and ADRD include differential DNA methylation of genes involved in metabolic regulation, immune signaling, and vascular function, as well as miRNA-mediated regulation of mitochondrial and synaptic pathways. These alterations are influenced by cumulative environmental and social exposures, including chronic stress, dietary factors, and comorbid cardiometabolic disease. Together, these findings support a model in which epigenetic mechanisms act as integrators of environmental and societal influences, contributing to heterogeneity in disease onset and progression across racioethnic groups [15,30,64]. Indigenous populations remain critically underrepresented in ADRD research. Recent work examining miRNA profiles in the Marshallese population residing in the United States identified differential expression of multiple miRNAs associated with chronic disease risk, highlighting the relevance of epigenetic regulation in this historically marginalized community. Although cognitive outcomes were not the primary focus, these findings underscore the importance of extending epigenomic research to Indigenous populations to better understand ADRD risk in culturally and environmentally distinct contexts [69].

Qin et al. (2020) conducted a study where they examined genome-wide profiles of both 5mC and 5hmC in the frontal cortex tissues of late-onset Chinese AD patients and cognitively normal individuals [70]. This study marked the first comprehensive analysis of DNA hydroxymethylation in the frontal cortex of AD patients in China, highlighting the importance of 5hmC in AD development. Their findings revealed unique alterations in the brain’s hydroxymethylome specific to the Chinese population, as well as some similarities with Caucasian cohorts. Comparison with Caucasian data showed distinct Chinese-specific and shared differentially hydroxymethylated regions (DhMRs), indicating the significant involvement of 5hmC in AD pathology. Notably, differences in 5hmC changes between Chinese and Caucasian cohorts underscored the population-specific epigenetic variations associated with AD. Additionally, the study identified common and ethnicity-specific pathways that could be epigenetically disrupted, contributing to AD onset. Notably, pathways related to glucose and lipid metabolism were prominently affected among the Chinese-specific DhMRs, suggesting a potential role of altered DNA hydroxymethylation in genes linked to these pathways in AD development in China [70].

Morris et al. (2019) conducted a study involving 1255 participants from various racial backgrounds. This longitudinal study spanned from 2004 to 2015 utilized magnetic resonance imaging of the brain, positron emission tomography with Pittsburgh compound B, and cerebrospinal fluid (CSF) assays to measure concentrations of amyloid-β42, total tau, and phosphorylated tau181. Pittsburgh compound B serves as a radioligand for aggregated amyloid-β. The researchers determined the Apolipoprotein E genotype of all individuals using genotyping rs7412 and rs429358 for APOE ε2, ε3, and ε4 isoforms. The findings revealed significant differences in CSF concentrations of tau protein between African American and white individuals, indicating potential race-specific biological mechanisms influencing the expression of AD among different racial groups [14].

Kalewold (2020) suggest that the existence of racial disparities in various biomedical outcomes, including AD, chronic kidney disease, and low birth weights, highlights the continued importance of race in understanding and potentially addressing epidemiological differences among different racial groups in the United States [71]. Kalewold further notes two challenges in exploring the causes and potential solutions to epidemiological racial inequalities, which stem from differences in illness rates among self-identified racial groups [71,72,73].

## 7. Conclusions and Future Directions

Epigenetic mechanisms represent promising therapeutic targets in AD and ADRD. Histone deacetylase (HDAC) inhibitors, DNA methylation modulators, and miRNA-based therapies have shown promise in preclinical models by improving synaptic plasticity, reducing neuroinflammation, and restoring transcriptional balance. However, emerging evidence suggests that epigenomic landscapes vary across populations due to differential environmental exposures and life-course stressors. As a result, therapeutic efficacy may differ across racioethnic groups, underscoring the importance of incorporating population-specific epigenomic profiling into therapeutic development and clinical trial design.

In the context of racial and ethnic disparities, epigenetic therapies may have differential efficacy due to population-specific epigenomic landscapes shaped by cumulative environmental exposures, social stressors, and comorbid conditions. For example, DNA methylation modulators and histone deacetylase (HDAC) inhibitors may target pathways related to inflammation, metabolism, and synaptic plasticity that are differentially regulated across populations. Similarly, miRNA-based therapeutic strategies—particularly those targeting mitochondrial and neuroinflammatory pathways may require population-specific validation to account for baseline differences in miRNA expression profiles. These considerations highlight the need for inclusive preclinical and clinical studies to ensure that emerging epigenetic therapies are both effective and equitable.

Racial and ethnic minority populations remain substantially underrepresented in AD/ADRD clinical trials. Barriers to participation include historical mistrust of biomedical research, limited access to specialty care, language barriers, socioeconomic constraints, and insufficient representation of minority investigators within research teams. This underrepresentation limits the generalizability of biomarker discovery and therapeutic response, thereby constraining progress toward equitable precision medicine. Randomized clinical trials (RCTs) are rigorous and tightly regulated processes aimed at translating scientific hypotheses into therapeutic advancements [74]. To be effective and targeted, they must include groups that stand to benefit the most from the outcomes. RCTs often require large sample sizes, ranging from hundreds to thousands of participants, and significant time commitments from patients to identify meaningful differences from control conditions, especially for treatments aimed at altering the course of a disease. The participants who meet the study criteria in standard parallel group RCTs are randomly assigned to one of several groups, and the outcomes are compared between these groups. It has been noted that RCTs often exclude around 75% of potential participants and eventually enroll only about 10% of those with the condition of interest. This strict selection process is especially prevalent in clinical trials for AD [75]. These structural limitations disproportionately affect racial and ethnic minority populations.

This underrepresentation is concerning, particularly given the projected growth in minority populations in the United States and Canada. Therefore, to ensure the relevance and efficacy of trials, it is imperative to increase the enrollment of racial minorities in AD RCTs. While there has been extensive discussion on the lack of recruitment in these populations, much of the focus has been on the African American and Latino communities, with less attention given to Indigenous peoples. Furthermore, some recruitment-related studies are not specific to AD [76]. Improving engagement with underrepresented communities through community-engaged research, transparent communication, culturally tailored recruitment strategies and increased representation within research teams will be critical for improving trial participation and outcomes.

**Figure 2:**
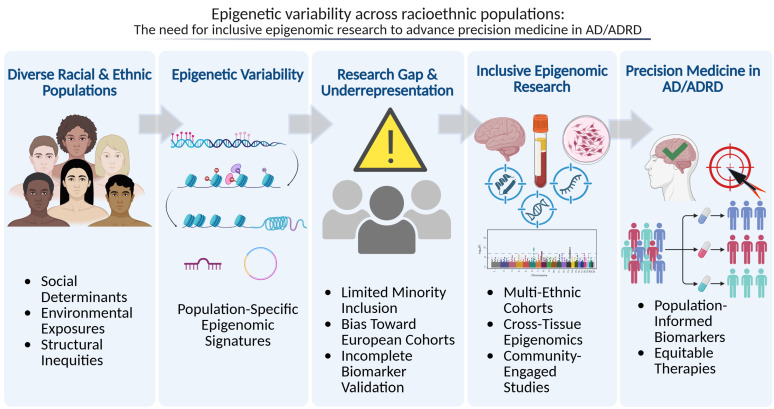
Conceptual framework illustrating how epigenetic variability across racioethnic populations underscores the need for inclusive epigenomic research to advance precision medicine in AD/ADRD. Diverse racial and ethnic populations experience distinct social, environmental, and structural exposures that contribute to population-specific epigenomic variation, including differences in DNA methylation, histone modifications, and non-coding RNA regulation. Underrepresentation of minority populations in epigenomic studies limits biomarker discovery and validation, leading to gaps in translational research. Inclusive epigenomic approaches incorporating multi-ethnic cohorts, cross-tissue profiling, and community-engaged research are essential for developing population-informed biomarkers and equitable therapeutic strategies for AD and ADRD.

Figure 2 provides a summative framework illustrating how epigenetic variability across racioethnic populations, coupled with underrepresentation in research, contributes to disparities in AD and ADRD and underscores the need for inclusive epigenomic approaches to advance precision medicine.

Future research recommendations emerging from this review include: (i) prioritizing the inclusion of racially and ethnically diverse populations in epigenetic biomarker discovery studies; (ii) validating epigenetic biomarkers, including DNA methylation signatures and non-coding RNAs, across multiple populations to ensure generalizability; (iii) integrating epigenetic endpoints into longitudinal cohort studies and randomized clinical trials; and (iv) adopting community-engaged and culturally informed research frameworks to improve recruitment, retention, and interpretation of epigenetic data in underrepresented populations. Together, these approaches are essential for translating epigenomic insights into equitable precision medicine strategies for AD and related dementias.

## Figures and Tables

**Table 1:** Dysregulated miRNAs in Alzheimer’s disease and related dementias.

miRNA	Sample Type	Localization	Reported Association	Reference(s)
miR-122-5p	Neuronal EVs, plasma	Neuron-derived	Biomarker potential; metabolic and inflammatory pathways	[60]
miR-125b	Brain, CSF	Neuronal	Tau phosphorylation, synaptic dysfunction	[64]
miR-135a	Blood, CSF, brain	Systemic/neuronal	Cognitive decline; replicated across tissues	[64]
miR-146a	Brain, CSF, blood	Microglia-associated	Neuroinflammation, immune signaling	[64,66,67]
miR-193b	Plasma exosomes/serum	Circulatory	Potential AD biomarker	[66]
miR-16	CSF/plasma	Circulatory	Regulators of β-amyloid processing	[66]
miR-501-3p	CSF exosomes	Exosomal	Upregulated in AD exosomes	[68]
miR-34a	Brain	Neuronal	Aging, mitochondrial dysfunction	[67]

## Data Availability

No new data were created or analyzed in this study. Data sharing is not applicable to this article.

## Abbreviations

The following abbreviations are used in this manuscript:

AD	Alzheimer’s disease
ADRD	Alzheimer’s disease and related dementias
CSF	Cerebrospinal fluid
RCTs	Randomized clinical trials
GWAS	Genome-wide association studies
VMR	Variably methylated region
VMP	Variably methylated probe
STG	Superior temporal gyrus
Aβ	Amyloid beta
NFT	Neurofibrillary tangle
ROS	Reactive Oxygen Species
MFG	Middle frontal gyrus
ncRNA	Noncoding RNA
circRNA	Circular RNA
DhMR	Differentially hydroxymethylated region
lncRNA	Long non-coding RNA
mRNA	Messenger RNA
miRNA	microRNA
